# Analysis of Silver Alert Reporting System Activations for Missing Adults With Dementia in Texas, 2017 to 2022

**DOI:** 10.1001/jamanetworkopen.2022.55830

**Published:** 2023-02-13

**Authors:** Anthony D. McDonald, Valerie Danesh, John M. Ray, Alan B. Stevens

**Affiliations:** 1 Department of Industrial and Systems Engineering, University of Wisconsin, Madison; 2 Center for Applied Health Research, Baylor Scott & White Health, Dallas, Texas; 3 Institute for Law Enforcement and Protective Services Excellence, Texas A&M Engineering Extension Service, College Station

## Abstract

This cross-sectional study analyzes data from Silver Alert activations in Texas from 2017 to 2022 to identify temporal, geographic, and wandering characteristics of missing adults with dementia.

## Introduction

Older adults with Alzheimer disease and related dementias are at high risk of wandering, leading to harm, accidental death, and care partner stress.^1,2^ Community-aided immediate search activities are often successful at finding missing individuals with dementia.^3,4^ Silver Alert programs are community-level, police-activated notification systems consisting of informational broadcasts to locate missing adults with dementia.^3^ The Texas network of missing-person alerts includes broadcasts via media, lottery retailers, and state transportation and public safety departments to elicit tips to law enforcement. Highway sign activation is included when vehicle information is known. We analyzed Silver Alert activations in Texas to identify temporal, geographic, and wandering characteristics of missing adults with dementia.

## Methods

Because this cross-sectional study used publicly available data, the Baylor Scott & White Research Institute Institutional Review Board designated it exempt and waived informed consent. We followed the STROBE reporting guideline.

Data were obtained from the Texas Department of Public Safety under a US Freedom of Information Act request for Silver Alerts issued between August 8, 2017, and March 9, 2022. Data were sourced from report logs of Silver Alert activation dates and times, demographic characteristics, last known location, time of last sighting, and vehicle characteristics. All demographic data were generated by the Silver Alert reporter.

County population estimates were drawn from 2020 US Census Bureau data. Poisson regression was used to compare silver alert rates by day of the week. Regression and correlation analyses were used to describe associations among characteristics. Statistical analysis was conducted using R, version 4.0.4 (R Project for Statistical Computing). Additional details are provided in the eMethods in Supplement 1.

## Results

We analyzed data from 548 Silver Alert activations, representing 524 missing adults with dementia (Table). Their median age was 77.0 years (IQR, 72.0-82.0 years); 144 were women (37.9%) and 380 were men (72.5%). Race and ethnicity were reported as Asian or Pacific Islander (14 [2.7%]), Black (101 [19.3%]), Hispanic (unknown race; 49 [9.4%]), White (358 [68.2%]), or other (2 [0.4%]).

**Table.** Demographic and Silver Alert Characteristics of Missing Adults With Dementia

Characteristic	No. of adults (%)		
	Total (N = 524)	Without a missing vehicle (n = 194)	With a missing vehicle (n = 330)
Age, y			
Median (IQR)	77.0 (72.0-82.0)	74.7 (69.3-80.0)	79.0 (74.0-82.0)
<65	18 (3.4)	10 (5.1)	8 (2.4)
65-74	150 (28.6)	82 (42.2)	68 (20.6)
75-84	263 (50.2)	78 (40.2)	185 (56.1)
≥85	93 (17.7)	24 (12.4)	69 (20.9)
Sex			
Female	144 (37.9)	67 (34.5)	77 (23.3)
Male	380 (72.5)	127 (65.5)	253 (76.7)
Race and ethnicity^a^			
Asian or Pacific Islander	14 (2.7)	4 (2.1)	10 (3.0)
Black	101 (19.3)	59 (30.4)	42 (12.7)
Hispanic, race unknown	49 (9.4)	28 (14.4)	21 (6.4)
White	358 (68.2)	101 (52.1)	257 (77.9)
Other	2 (0.4)	2 (1.0)	0
Vehicle type (n = 338)			
Sedan	NA	NA	145 (43.9)
Sport utility vehicle	NA	NA	96 (29.1)
Truck	NA	NA	82 (24.8)
Van	NA	NA	15 (4.5)
Motorcycle	NA	NA	0
Activation, time of day (n = 548)			
Day (7:00 am to 2:59 pm)	76 (13.9)	33 (16.3)	43 (12.4)
Afternoon/evening (3:00 to 10:59 pm)	234 (42.7)	79 (39.1)	155 (44.8)
Night (11:00 pm to 6:59 am)	238 (43.4)	90 (44.5)	148 (42.8)
Activation on a holiday (n = 548)	139 (26.5)	67 (34.5)	72 (21.8)
Silver Alert outcome (n = 548)			
Active	1 (0.1)	1 (0.1)	0
Discontinued	33 (6.0)	17 (3.1)	16 (2.9)
Found	514 (94.7)	184 (33.6)	330 (60.2)

Abbreviation: NA, not applicable.

^a^ Race and ethnicity reporting are based on data definitions by the Texas Department of Public Safety, which do not provide specifics for the “other” category.

Temporal trends illustrated year-over-year increases in Silver Alert activations (16.0%) (Figure, A). The number of events by day peaked on Wednesdays (108 [19.7%], β = 0.59, *P* < .001) (Figure, B). The number of events by hour peaked at 10 pm (42 [7.6%]) and 3 am (40 [7.3%]), with a smaller peak at 6 pm (35 [6.4%]) (Figure, C). Substantially more Silver Alert events occurred on holidays, with 14 holidays (3.8% of the 365-day calendar year) representing one-quarter of all Silver Alerts (139 [25.4%]). Geographically, activations were generally proportionate to population density by county.

**Figure.**
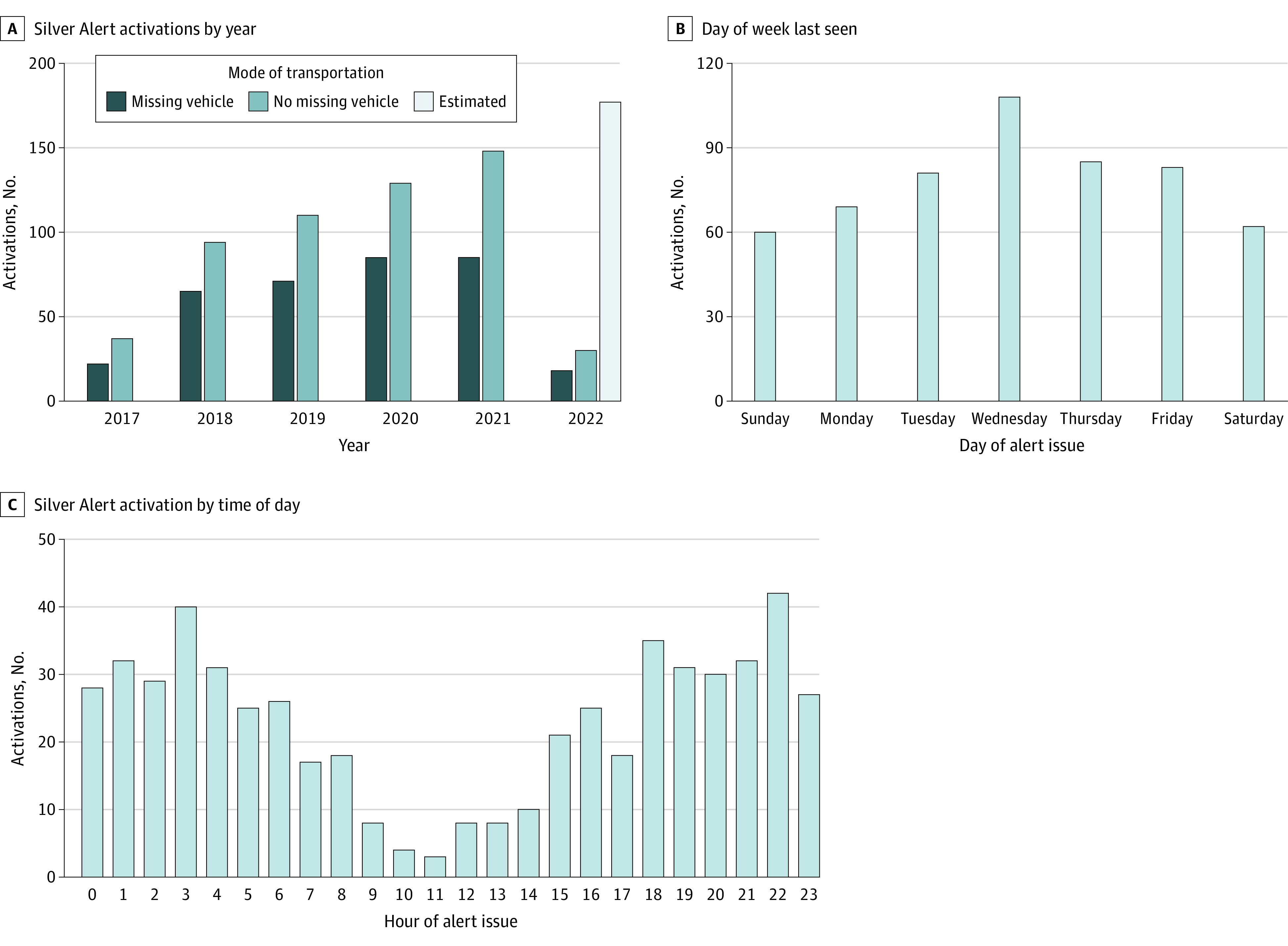
Temporal Characteristics of Silver Alert Activations in Texas by Year, Day of Week, and Time of Day, 2017 to 2022 A, The number of Silver Alert activations increased every year, and the majority had a vehicle involved. Activations in 2017 represent August 8 to December 31, 2017, and activations in 2022 represent January 1 to March 9, 2022. B, The number of silver alerts was highest on Wednesdays. C, The number of silver alerts peaked at 10 pm.

Most Silver Alert activations (338 [61.7%]) included a missing vehicle. Most alerts (145 [43.9%]) were issued for drivers of sedans (Table), and most vehicle model years (median, 2012 [range, 1988-2021]) were within 8 years of the activation date. Age and sex (odds ratios, 1.08 [95% CI, 1.05-1.11] and 1.71 [95% CI, 1.14-2.57]; both *P* < .001) were associated with vehicle involvement in silver alerts, with male and older individuals more likely to be driving a vehicle compared with female and younger individuals, respectively.

## Discussion

In this cross-sectional study, demographic, temporal, geographic, and vehicle-related trends among Silver Alert activations suggested that there were nonrandom variations in Texas, with year-over-year increases in activations outpacing the state population-level increases of older adults (16.0% vs 3.8%). Most vehicles involved in Silver Alerts were model years 2012 or newer. Newer models may increase opportunities for older adults to use in-vehicle navigation.^5^

This study had the following limitations: community-managed and unreported cases were excluded; the analyses were correlational and should be interpreted with caution; and the data set had limited outcomes (eg, found alive or deceased). Future research could include sourcing data from narrative reports or interviews with care partners and police officers. Our state-specific sample limits the generalizability of our findings, but associations of Silver Alerts with the involvement of vehicles and older male individuals—as well as activation peaks on Wednesdays, at night, and on holidays—reflect important considerations for care partners and clinicians involved in the care of older adults.

## Notes

Supplement 1.
eMethods.

eReferences

Supplement 2.
Data Sharing Statement
