# Supplementary material for: Analysis of Silver Alert Reporting System Activations for Missing Adults With Dementia in Texas, 2017 to 2022
Source: JAMA Netw Open. 2023 Feb 13;6(2):e2255830. doi: 10.1001/jamanetworkopen.2022.55830 (PMC9926327; doi:10.1001/jamanetworkopen.2022.55830)
Supplement: Supplement 1. — eMethods. eReferences [file jamanetwopen-e2255830-s001.pdf]

Supplementary Online Content

McDonald AD, Danesh V, Ray JM, Stevens AB. Analysis of Silver Alert reporting system activations for missing adults with dementia in Texas, 2017 to 2022. *JAMA Netw Open*. 2023;6(2):e2255830. doi:10.1001/jamanetworkopen.2022.55830

eMethods.

eReferences

This supplementary material has been provided by the authors to give readers additional information about their work.

eMethods

Data collection, population, and settings

Data was collected retrospectively from the Texas Department of Public Safety under a U.S. Freedom of Information Act request for silver alerts. Data included dates and times of Silver Alert activations, with demographics and time and geographic location last seen reported by the family or guardian of the missing adult with dementia, including identifying vehicle information when applicable. The administrative data for silver alert reports are sourced from state records of driver's licenses and from the silver alert reporter. There were no missing data for age, race, ethnicity, geographic location last seen or identifying vehicle information when applicable. No sample size was calculated because all events between August 8, 2017 – March 9, 2022 were included.

Measures

Silver Alert data were inspected to remove duplicates and derive additional variables, including temporal classifications of dates and times (e.g., day of week, holiday) and vehicle type classifications drawn from make and model information (e.g., truck, sedan). Vehicle involvement was defined as the inclusion of vehicle information with the alert, which is consistent with Texas procedures for inclusion only when the missing adult with dementia is associated with the known and missing vehicle. Holidays were defined using the isHoliday and holidayDate functions in the TimeDate package in R[1-2]. US federal and Christian holidays were included. The inclusion of US federal and Christian holidays was guided by school observances and the demographics of the state of Texas.

© 2023 McDonald AD et al. *JAMA Network Open*.

Statistical analysis

Data are expressed as median (IQR) or frequency (percentage) as appropriate. Poisson regression was used to model silver alert activation rates per day of week. Logistic regression was used to model silver alerts with vehicle involvement, silver alerts without vehicle involvement, and all silver alerts, controlling for a number of other factors including driver age, biological sex, and calendar year. Data analysis and visualizations were conducted in R (version 4.0.4) using tidyverse, stats, and data.table[1-4].

Quality control

The dataset was reviewed by multiple authors to ensure accuracy.

© 2023 McDonald AD et al. *JAMA Network Open*.

eReferences

1. R Core Team. R: A Language and Environment for Statistical Computing. R Foundation for Statistical Computing. https://www.R-project.org/. Published 2018. Accessed.
2. _*timeDate: Rmetrics - Chronological and Calendar Objects*. Version R package version 4021.1072022.
3. Wickham H, Averick M, Bryan J, Chang W, McGowan LD, François R, Grolemund G, Hayes A, Henry L, Hester J, Kuhn M, Pedersen TL, Miller E, Bache SM, Müller K, Ooms J, Robinson D, Seidel DP, Spinu V, Takahashi K, Vaughan D, Wilke C, Woo K, Yutani H (2019). “Welcome to the tidyverse.” _Journal of Open Source Software_, 4(43), 1686. doi: 10.21105/joss.01686.
4. Dowle M, Srinivasan A (2022). _data.table: Extension of `data.frame`_. R package version 1.14.6, https://CRAN.R-project.org/package=data.table.

© 2023 McDonald AD et al. *JAMA Network Open*.
