# Supplementary material for: Analysis of Silver Alert Reporting System Activations for Missing Adults With Dementia in Texas, 2017 to 2022
Source: JAMA Netw Open. 2023 Feb 13;6(2):e2255830. doi: 10.1001/jamanetworkopen.2022.55830 (PMC9926327; doi:10.1001/jamanetworkopen.2022.55830)
Supplement: Supplement 2. — Data Sharing Statement [file jamanetwopen-e2255830-s002.pdf]

Data Sharing Statement

McDonald. Analysis of Silver Alert Reporting System Activations for Missing Adults With Dementia in Texas, 2017 to 2022. *JAMA Netw Open*. Published February 13, 2023. doi:10.1001/jamanetworkopen.2022.55830

Data
Data available: Yes
Data types: Deidentified participant data, Data dictionary
How to access data: Due to the sensitive information at the participant level, the authors cannot take direct responsibility for making data publicly available. Access to the data will require an executed Data Sharing Agreement between the requestor and Baylor Scott & White Research Institute.
When available: With publication

Supporting Documents
Document types: None

Additional Information
Who can access the data: Anyone requesting the data for research-only use.
Types of analyses: Research analyses to contribute to peer-reviewed health services or patient outcomes research.
Mechanisms of data availability: To begin the request process contact the corresponding author of the manuscript.
